# The Y.M.C.A. Anti-Tuberculosis Campaign

**Published:** 1917-09-15

**Authors:** 


### THE Y.M.C.A. ANTI-TUBERCULOSIS CAMPAIGN.

### An Educational Biograph Film.

The National Council of Young Men's Christian Associations intends to organise a series of film-illustrated lectures concerning tuberculosis in the huts behind the lines at the Western seat of war. The Y.M.C.A. in its war work quickly perceived a unique opportunity and, grasping it in a manner beyond praise, has done a work which national gratitude should never allow to be forgotten; it has, moreover, acquired a social influence the strength of which may prove later to be of the greatest value. The Association, which believes in a co-ordination of the functions of priest and physician, has already interested itself in the "consumptive" and (adopting advanced views) in the farm colony as a place for treatment.

The educational campaign which is now to be commenced was inaugurated by a meeting held at the King George's Hall, London Central Y.M.C.A., Tottenham Court Road, on Tuesday, September 4, under the chairmanship of the Rev. Archibald Fleming, D.D. At this meeting a lecture was given by Dr. Halliday Sutherland on the cure and eradication of tuberculosis on the Edinburgh system and the film, "The Story of John M'Neil," presented to the Association by the Royal Victoria Trust of Edinburgh, was shown. Dr. Halliday Sutherland, who has done valuable work at the St. Marylebone Tuberculosis Dispensary, gave his audience a most instructive outline of the history, causes, dangers, and treatment of tuberculosis, laying stress upon the economic loss resulting from the prevalence of the disease and upon the duty of the State in its prevention. He emphasised the extraordinary supineness which has been shown as regards the control of our milk-supply. He pointed out the need for steadying public opinion, which, after unduly vaunting sanatorium treatment and being disappointed in its immediate results, may in the next decade imagine that it sees a panacea in the open-air school.

### Points that Require Emphasis.

Dr. Sutherland's address was carefully worded and ably delivered. It is the less disagreeable task to express criticism of it because its own chief fault lay in a tendency harshly and censoriously to criticise our national characteristics in general and the failings, or supposed failings, of our Government Departments and public bodies in particular. We agree that it is unfortunate that the Milk and Dairies Act of 1914 cannot be brought into force until after the war, but we think that it is better so than to have it nominally effective but in practice largely ignored, as it assuredly would have been. Dr. Sutherland was dogmatic, perhaps he was intentionally so as he was addressing a lay audience: some of his statements as to the carriage of infection and the disinfection of sputum are, however, founded on personal rather than on accepted views. Sound deductions as to the influence of hereditary predisposition in tuberculosis cannot be founded upon the results of the examination of children. The importance of the housing question was hardly given full value. Finally, so full a lecture might usefully have contained some reference to auto-inoculation, which can be brought within the comprehension of, and made very interesting to, an intelligent audience. The matter is important because the belief is, even yet, not uncommon that, in being given graded work during sanatorium treatment, patients are being exploited as gardeners or navvies for the benefit of the institution.

The film-story of John M'Neil is a well-conceived exposition of many points in connection with tuberculosis and its treatment; it is intended, and is highly suitable, for popular exhibition. The M'Neil family, in their stuffy tenement in Edinburgh, become subject to tuberculous infection. The elder girl seeks medical advice, thereby bringing the machinery of the dispensary into action to deal with the whole family. The faults of the film are mainly those of the cinema and its methods; the scene of the visit of the nurse to the home of the M'Neils is given with that ludicrous impression of unseemly and tremulous haste with which picture-palace frequenters are no doubt, in indoor scenes, familiar. Then we see Mrs. M'Neil hurried (it is the only appropriate word) to the hospital for advanced cases, "where her symptoms are relieved and her life is brightened," and where she evidently dies. Upon the latter point the story is, perhaps wisely, reticent, although there is a suggestion of heartlessness about the way in which the family is rid of her.

### A Missed Opportunity.

Incidentally it may be remarked that an important need in the provision of hospital treatment for tuberculous (as, indeed, for other) patients is reasonable facility for visiting by relatives and friends. Patients and their friends are prone to think that their feelings in this matter are ignored, and even that visiting is intentionally discouraged. It would have added to the sympathetic interest of the film had M'Neil and his children been shown visiting Mrs. M'Neil in one of the balconies of the hospital or in the revolving shelter, which is shown occupied by two men whose antics are more droll than was probably intended. A scene depicting a visit to Mrs. M'Neil need have had no suggestion of sickly sentimentality or even of pathos. We are next shown the younger children at an open-air school and M'Neil at the sanatorium, where he shares in graded work of various kinds before being transferred to a farm colony: there, in some of the best pictures of the series, he is shown gardening, feeding pigs and poultry, road-making, etc. Having obtained open-air employment he is rejoined, in a new and brighter home, by his children.

